# Two-stage PCR assay for detection of human brucellosis in endemic areas

**DOI:** 10.1186/1471-2334-13-145

**Published:** 2013-03-21

**Authors:** Ibrahim Hassan Kamal, Basim Al Gashgari, Said Salama Moselhy, Taha Abdullah Kumosani, Khalid Omar Abulnaja

**Affiliations:** 1 Biochemistry Department, Faculty of Science, King Abdulaziz University, Jeddah, Saudi Arabia; 2 Biochemistry Department, Faculty of Science, Ain Shams University, Cairo, Egypt; 3 Experimental Biochemistry Unit, King Fahd Medical Research Center (KFMRC), King Abdulaziz University, Jeddah, Saudi Arabia

## Abstract

**Background:**

Brucellosis is a common zoonosis that can cause a severe febrile illness in humans. It constitutes a persistent health problem in many developing countries around the world. It is one of the most frequently reported diseases in Saudi Arabia and incidence is particularly high in the Central region, and around the city of Riyadh. The aim of this study was to evaluate a two-stage PCR assay for detection of human brucellosis particularly in endemic areas.

**Methods:**

A total of 101 serum samples were collected from patients with acute febrile illness (AFI) of unknown cause from two different locations in the Western region of Saudi Arabia. The first location (Northern) is characterized by a nomadic rural population while the second (Central) is a modern urban city. All samples were subjected to DNA extraction and *Brucella* genus-specific PCR amplification using B4/B5 primers of the *bcsp31* gene. Positive B4/B5 samples were subjected to multiplex species-specific *Brucella* PCR amplification.

**Results:**

In the Northern location, 81.9% of the AFI samples were confirmed *Brucella* positive, while all the samples collected from the Central region proved to be *Brucella* negativ*e*. Samples positive for *Brucella* were subjected to multiplex species-specific *Brucella* amplification. *B. abortus* was detected in 10% and *B. melitensis* in 8% of the samples, while the majority (82%) of samples showed both *B. abortus* and *B. melitensis*. As expected, *B. suis* was not detected in any of the samples.

**Conclusions:**

This study concluded that a two-stage PCR assay could be useful as a rapid diagnostic tool to allow the consideration of brucellosis as a possible cause of AFI, particularly in non-urban locations. It also recommends the collection of epidemiological data for such patients to obtain further information that may help in rapid diagnosis.

## Background

The etiology and incidence of acute febrile illness (AFI) represents a major public health problem because clinical diagnosis is usually unreliable, and diagnostic tests are often not available in disease endemic areas [1]. Surveillance based on symptoms alone frequently results in classification errors, because febrile illnesses resulting from different pathogens may be clinically indistinguishable. Ideally a good surveillance system should be supported by modern molecular diagnostic tests and be sensitive and specific enough to accurately reflect the causes of febrile illnesses in a population. Brucellosis is a severe acute febrile disease caused by Gram-negative bacteria of the genus *Brucella*. It is the cause of a wide range of significant veterinary and public health problems, and economic loss [2]. The eradication of human brucellosis is difficult and the disease has a serious medical impact worldwide [3]. The acute febrile clinical symptoms of brucellosis always overlap with those of other etiological pathogens, and this may lead to misdiagnosis as well as improper antibiotic treatment regimes.

Human brucellosis is one of the most frequently reported diseases in Saudi Arabia, particularly in the Central region and around the city of Riyadh [4-10]. Since brucellosis is a zoonotic disease, it is transmitted from animals to humans by direct contact with infected animals or consumption of raw animal products such as unpasteurized milk or cheese. Direct contact with infected animals, their secretions or their carcasses can lead to infection through inhalation or accidental skin and mucous membrane penetration [11,12]. In Saudi Arabia, brucellosis has been recognized as a major health problem, and measures to control the disease were implemented as early as 1983 [13].

Four species of the genus *Brucella* are pathogenic for humans, namely *B. melitensis* (from sheep and goats), *B. abortus* (from cattle and other bovidae), *B. suis* (from pigs), and *B. canis* (from dogs) [14]. Queipo-Ortuno and coworkers [15] found 100% sensitivity and 98.3% specificity using the B4/B5 primer pair amplifying a 223 bp fragment of the *bcsp31* gene, compared with 70% sensitivity for diagnosis by blood culture. PCR identification of *Brucella* strains at the species or biovar level has been described by Redkar *et al.*[16], who developed a real-time PCR assay for the detection of *B. abortus*, *B. melitensis*, and *B. suis* biovar 1. These PCR assays target the specific integration of IS*711* elements within the genome of the respective *Brucella* species or biovar.

In most developing countries, especially in non-urban areas, real-time PCR facilities are not available as a diagnostic tool for human brucellosis. Most diagnostic laboratories still rely on routine laboratory tests such as bacterial culturing and serological tests, even though thermal cyclers may be available. In this study, we took the initiative to evaluate a two-stage PCR assay as a rapid sensitive diagnostic tool for diagnosis of human brucellosis, to highlight the need to consider brucellosis in the differential diagnosis of AFI, particularly in non-urban areas where patients are known to have a risk of exposure and *Brucella* incidence is expected to be high.

Two different locations in the Western region of Saudi Arabia were selected to test the two-stage PCR strategy. The Northern location is characterized by a nomadic, mostly Bedouin population who consume unpasteurized dairy products and ingest fresh camel, goat and sheep milk. The Central location was in a modern urban city where the Bedouin population is low and the chances of using unpasteurized dairy products or direct exposure to animals are limited. These two locations were selected to test the likelihood of *Brucella* infection being a major cause of AFI especially in rural locations, where the lifestyle of the population allows contact with *Brucella*-infected animals.

## Methods

### Subjects

A total of 101 serum samples were collected from the two selected locations in the Western region of Saudi Arabia. In the Northern region, samples were from the Armed Forces hospital of Tabuk during the period June 2009 to January 2011, and in the Central region samples were collected at King Abdulaziz Hospital of Jeddah during the period November 2009 to November 2011. Serum was obtained from patients aged 1 year or older suffering from unknown fever of more than 2 days’ duration who sought medical help in hospital. AFI was defined as a body temperature ≥ 38°C at the time of collection, or fever of more than 2 days, and no other identified cause of fever such as diarrhea, hepatitis or any respiratory tract infections. Control serum samples were collected from 20 healthy volunteers from the same locations. No family history or any occupational exposure to *Brucella* infection was recorded for the healthy controls. All participants meeting inclusion criteria were asked to participate in this study. Informed written consent was obtained from adult participants and parents of minors.

All samples were subjected to DNA extraction as previously described [17], with minor modifications as follows: 1% of sodium dodecyl sulfate (SDS) and 10 mg/ml of proteinase K were added to 300 ul of serum and incubated for 2 h at 37°C. Proteinase K in the digest was inactivated by heating at 90–95°C for 10–l5 min. After phenol-chloroform-isoamyl alcohol extraction and ethanol precipitation, DNA was dissolved in 50 ul of nuclease-free water.

### *Brucella* genus-specific DNA amplification

To diagnose the *Brucella* positive samples, the first PCR amplification was carried out using primers designed to target a 223 bp fragment of the *bcsp31 gene.* This sequence encodes an immunogenic membrane protein of a 31 kDa antigen of *B. abortus* and is conserved in all *Brucella* biovars [18]. A pair of 21-nucleotide primers, B4 (5^′^ TGG CTC GGT TGC CAA TAT CAA 3^′^) and B5 (5^′^ CGC GCT TGC CTT TCA GGT CTG 3^′^), were obtained from Bioline, Inc., (Taunton, MA, USA), as described by Baily *et al.*[19]. PCR was performed in a 25 ul mixture containing template DNA; PCR buffer (10 mM Tris HCl [pH 8.4], 50 mM KCl, 1.5 mM MgCl_2_); 10 pmol of each primer; 200 uM (each) of dATP, dCTP, dTTP and dGTP (Bioline, Inc.), and 1.25 U of *Taq* polymerase (Qiagen, Chatsworth, NJ, USA). The cycle consisted of a preheating step at 95°C for 5 min followed by 35 cycles of 90°C for 1 min, 60°C for 30 s, and 72°C for 1 min with a final incubation at 72°C for 10 min. A positive control based on DNA from a *B. abortus* reference strain was included in all tests, as well as a negative control containing all of the components of the reaction mixture except DNA. 20% of each PCR product was visualized on a 1% agarose gel stained with 2 ug/ml of ethidium bromide.

### Multiplex species-specific *Brucella* DNA amplification

All samples positive using the B4/B5 primers were subjected to multiplex PCR to determine which *Brucella* species might be causing the infection. Species-specific DNA segments of *B. abortus*, *B. melitensis* and *B. suis* were targeted for amplification using specific primers derived from the *IS*711 element [20]. The forward primer (5^′^ CAT GCG CTA TGT CTG GTT AC 3^′^) spans 803 to 823 nt of *IS*711 and generates a 113 bp PCR product with *B. abortus* reverse primer (5^′^ GGC TTT TCT ATC ACG GTA TTC 3^′^), 252 bp PCR product with *B. melitensis* reverse primer (5^′^ AGT GTT TCG GCT CAG AAT AAT C 3^′^), and 170 bp product with *B. suis* reverse primer (5^′^ ACC GGA ACA TGC AAA TGA C 3^′^). Amplification conditions were the same as for the first PCR, except for the use of an annealing temperature of 58°C. Positive and negative PCR controls were used in all tests. *B. suis* primers were used as an internal negative PCR control. *B. suis* is pathogenic to pigs, which are not found in Saudi Arabia. PCR products were visualized on a 1% agarose gel as previously described.

## Results

A total of 101 AFI patients were enrolled in this study in the Western region of Saudi Arabia; 61 and 40 from the Northern and Central locations, respectively. Their characteristics are presented in Table 1. Forty-four samples (72%) from the Northern location were serologically positive for *Brucella* with varying titers (data not shown). All samples were subjected to *Brucella* genus amplification using B4/B5 primers that amplify a conserved region in all *Brucella* species to detect the presence of *Brucella* DNA as one of the possible causes of the AFI.

**Table 1:** Characteristic features of patients with AFI

**Characteristic**	**Values**	
	**Locality at Western region**	
	**Northern**	**Central**
No. of AFI samples collected	61	40
No. of *Brucella* seropositive samples (data not shown)	44/61	00/40
No. of samples subjected to conventional PCR (B4/B5) amplification	61/61	40/40
No. of samples subjected to species-specific PCR	50/61	00/40

Agarose gel electrophoresis of the B4/B5 conventional PCR amplification gave a product size of 223 bp, indicating the presence of *Brucella* genus in these patients. The PCR control of the *Brucella* reference strain amplified a product of a similar size. No amplification was detected in the negative PCR control, or in the negative control subjects. Conventional PCR confirmed that 50 samples (81.9%) were diagnosed as *Brucella* positive out of the 61 samples collected from the Northern location, leaving 11 (18%) patients whose AFI was of non-*Brucella* origin. No human brucellosis was detected in the 40 samples collected from the Central location.

**Figure 1:**
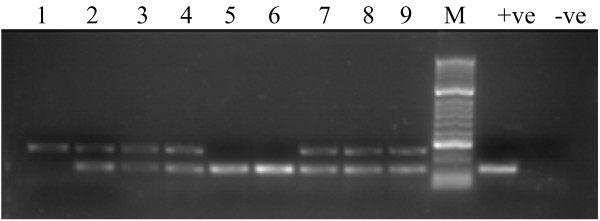
**Agarose gel electrophoresis of species-specific multiplex assay products.** Lane #1 *B. melitensis* only (252 bp). Lanes 5 and 6, *B. abortus* only (113 bp); lanes 2, 3, 4, 7, 8, and 9, both of *B. abortus* and *B. melitensis* (113 bp and 252 bp respectively). Lane (+ve) and (−ve) contain positive and negative PCR controls. Lane (M) contains a 100-bp ladder (HyperLadder, Bioline, Inc., MA, USA).

DNA from the 50 *Brucella* positive patients were subjected to the species-specific multiplex PCR. Multiplex PCR electrophoresis results are shown in Figure 1, which illustrates the presence of 113 bp and 252 bp bands specific for *B. abortus* and *B. melitensis*, respectively. Among the 50 samples, *B. abortus* alone was evident in five samples (10%) and *B. melitensis* alone in four samples (8%), while the rest of the samples (82%) showed products of both *B. abortus* and *B. melitensis* (Table 2). The *B. suis* amplification product (170 bp) was not detected in any of the samples. These results confirmed the specificity and sensitivity of these primers for the targeted region in *Brucella* DNA.

**Table 2:** ***Brucella *DNA amplification using B4/B5 and species-specific multiplex PCR**

**(AFI) Samples locality**	***Brucella *genus B4/B5 conventional PCR**		***Brucella *species-specific multiplex**		
**(Western region) (number)**	**-ve amplification**	**+ve amplification**	**Double product**	**Single product**	**Single product**
	**Non- *Brucella *patients**	***Brucella *patients**	***B. abortus & B. melitensis***	***B. abortus***	***B. melitensis***
(Northern region) (61)	11	50	41 (82%)	5 (10%)	4 (8%)
(Central region) (40)	40	-------	Not applicable		

## Discussion

AFI still represents a common clinical syndrome among patients seeking hospital care. *Brucella* is one of a number of pathogens causing febrile illness, and is a serious public health problem in many developing countries, including Saudi Arabia; where many people, by their traditional lifestyle, consume raw milk or have close animal contact [21]. The true human brucellosis incidence has been estimated to be between 10 and 25 times higher than the number of annual reported cases [22].

Diagnosis of human brucellosis in Saudi Arabia currently depends mainly on culture [23] and serological tests [24]. *Brucella* is a highly virulent bacterium and may constitute exposure hazards for laboratory personnel. Furthermore, its culture is time consuming and the isolation rate is low, which may cause critical diagnostic delays [25]. At the early stage of infection, the sensitivity of serologic tests is low and false-negative or only weak-positive reactions may occur [26]. Because of limitations of culture techniques and serological tests, various molecular methods, particularly PCR, have been developed for rapid identification of organisms in clinical samples. The PCR technique has proved to be a very useful, simple, quick, sensitive, specific and relatively inexpensive technique that merits its adoption in clinical laboratories. Several articles have been published dealing with various PCR-based methods for *Brucella* detection.

In this study, a two-stage PCR assay was tested. The genus-specific PCR assay, which targets the 223 bp sequence of the gene encoding a 31 kDa *Brucella abortus* antigen [19], and the multiplex amplification for the identification of *Brucella* to the species level called AMOS PCR for *B. abortus*, *B. melitensis*, *B. ovis* and *B. suis*[20], were carried out on 110 Saudi Arabian serum samples from patients with AFI. *Brucella* genus-specific B4/B5 primers detected the presence of the predicted 223 bp fragment in 81.9% of serum samples collected from the Northern location, but did not detect any *Brucella* cases out of the 40 samples collected from Central location. These results indicated that using serum as a clinical sample and the two PCR sequential assays provided a sensitive assay for diagnosis of human brucellosis. Similar results were reported by Elfaki and coworkers [27], who reported the presence of the same PCR fragment (223 bp) in 96% of the sera samples from 25 patients with symptoms of brucellosis from two reference hospitals in Central Saudi Arabia.

The *Brucella* species-specific multiplex PCR classified the *Brucella* genus positive samples into single *B. abortus* or *B. melitensis* or double infection (both *B. abortus* and *B. melitensis*), which represented the majority of cases (81%). *B. suis* primers failed, as predicted, since swine are not domestic in Saudi Arabia. Similar results were previously recorded in Saudi Arabia [27].

Our results support other studies [27,28], which recommended the use of PCR as the diagnostic tool of choice for human brucellosis. Since the samples in this study were collected randomly with very limited case histories, we could not classify the brucellosis as acute, chronic or relapsing cases.

The existence of the double product may be attributed to active double infection or to the coexistence of free DNA of one or both species in the tested samples. The free DNA in serum may reflect the degradation of *Brucella* cells during the bacteremic phase of infection [27]. Double infection may be attributed to keeping livestock of different species. The incidence of animal brucellosis in the Saudi Arabia Makkah region was previously found to be 0.8% in goats, 0.5% in sheep, 2.8% in camels and 3.6% in cows [29]. Ten years later in the Asir region, it had risen to 18.2% in goats, 12.3% in sheep, 22.6% in camels and 15.5% in cows [30].

Our results succeeded in highlighting the differences between the two selected locations in this study, where the non-urban area (Northern location) showed a high incidence of human brucellosis among the AFI patients, which could be due to probable exposure to infected dairy products or direct contact with infected animals common in the nomadic Bedouin population lifestyle in such rural locations. On the other hand, our assay failed to detect any brucellosis in samples collected from the urban city (Central location), where the Bedouin population is limited and exposure risk is low.

As mentioned by Dean and co-authors [31], health service inadequacies are compounded by socioeconomic factors, with brucellosis affecting poor, marginalized communities who often do not have the means to seek treatment. A study conducted in rural Tanzania revealed that 1 in 5 patients did not present to a health center for assessment until more than 1 year after the onset of illness. As a result of false-negative results, 44.8% brucellosis cases were not diagnosed at the hospitals on their first visit. These cases were treated for other diseases such as malaria, which is much more common in the rural area than brucellosis, and the brucellosis remained untreated [32]. Given the high proportion of brucellosis cases with fever, brucellosis should be considered as a differential diagnosis for fevers of unknown origin. Many patients from non-urban areas do not report to healthcare facilities.

## Conclusions

This study concluded that a two-stage PCR assay could be useful as a rapid diagnostic tool to highlight the need to consider brucellosis as a possible cause of AFI, particularly in non-urban locations.

The two-stage PCR assay minimizes exposure risks to laboratory personnel for this virulent bacterium and also shortens the diagnostic time. It may also be considered as an epidemiological tool for disease confirmation, tracing *Brucella* spp. transmission and identification of infection sources.

The study recommends that healthcare authorities determine the patient’s geographic location, lifestyle, age, gender, occupational exposure, food consumption, other health conditions (antibiotic treatment) and family history. This information plus species-specific diagnosis is useful for improving the diagnostic capacity, reducing the diagnostic delay, introducing new treatment regimens and providing strategies to effectively cure even the most complex cases of brucellosis often seen in endemic areas.

### Consent statement

Institutional ethical approval for the study was obtained from the Ethical Committee of King Abdulaziz University.

## Competing interests

The authors declared that they have no competing interests.

## Authors’ contributions

IHK designed and performed the study experiments and was responsible for writing the manuscript. BAG participated in the laboratory experiments. SSM, TAK and KOA were responsible for data management, and revised the draft carefully for important intellectual content. All authors read and approved the final version of this manuscript.

## Pre-publication history

The pre-publication history for this paper can be accessed here:

http://www.biomedcentral.com/1471-2334/13/145/prepub
